# Efficiency fosters cumulative culture across species

**DOI:** 10.1098/rstb.2020.0308

**Published:** 2022-01-31

**Authors:** T. Gruber, M. Chimento, L. M. Aplin, D. Biro

**Affiliations:** ^1^ Faculty of Psychology and Educational Sciences and Swiss Center for Affective Sciences, University of Geneva, Geneva, Switzerland; ^2^ Cognitive and Cultural Ecology Research Group, Max Planck Institute of Animal Behavior, Radolfzell, Germany; ^3^ Centre for the Advanced Study of Collective Behaviour, University of Konstanz, Konstanz, Germany; ^4^ Department of Biology, University of Konstanz, Konstanz, Germany; ^5^ Department of Zoology, University of Oxford, Oxford, UK; ^6^ Department of Brain and Cognitive Sciences, University of Rochester, Rochester, NY, USA

**Keywords:** social learning, cultural evolution, animal culture, efficiency, complexity

## Abstract

Recent studies in several taxa have demonstrated that animal culture can evolve to become more efficient in various contexts ranging from tool use to route learning and migration. Under recent definitions, such increases in efficiency might satisfy the core criteria of cumulative cultural evolution (CCE). However, there is not yet a satisfying consensus on the precise definition of efficiency, CCE or the link between efficiency and more complex, extended forms of CCE considered uniquely human. To bring clarity to this wider discussion of CCE, we develop the concept of efficiency by (i) reviewing recent potential evidence for CCE in animals, and (ii) clarifying a useful definition of efficiency by synthesizing perspectives found within the literature, including animal studies and the wider iterated learning literature. Finally, (iii) we discuss what factors might impinge on the informational bottleneck of social transmission, and argue that this provides pressure for learnable behaviours across species. We conclude that framing CCE in terms of efficiency casts complexity in a new light, as learnable behaviours are a requirement for the evolution of complexity. Understanding how efficiency greases the ratchet of cumulative culture provides a better appreciation of how similar cultural evolution can be between taxonomically diverse species—a case for continuity across the animal kingdom.

This article is part of a discussion meeting issue ‘The emergence of collective knowledge and cumulative culture in animals, humans and machines’.

## 1. Introduction

There are a growing number of examples of cultures [1] in non-human animals (henceforth animals), with such socially learned, group-specific patterns of behaviours found in a variety of phylogenetic taxa, including primates [2–4], birds [5,6], cetaceans [7–9] and insects [10]. Over the course of the last three decades, our understanding of these animal cultures has greatly benefited from the use of modelling [11–19], as well as field experiments [20–24] which have investigated social learning and culture directly in the animals' natural habitat, complementing controlled evidence from the laboratory [25]. However, very significant differences remain between the perception of human and animal cultures. For example, while some authors, often from a biological background, are content with a definition of culture based on socially transmitted traditions [1], others require more criteria such as particular social transmission mechanisms (e.g. imitation or teaching) [26,27]. Another large and more recent domain of debate has focused on the question of cumulative cultural evolution (CCE).

CCE has been defined and redefined by biologists, anthropologists and psychologists over the past three decades, creating many ambiguous criteria that constitute its definition. CCE was first proposed to contrast human and animal culture, as humans were able to add and retain modifications to cultural traits over time, dubbed the ratchet effect [28]. Included in this definition was the consequence that products of CCE would exceed what a single individual could innovate alone within a lifetime [29]. Further, CCE has been proposed to rely on possibly human-unique social learning mechanisms, such as imitation [26,30,31], although the importance of these mechanisms is under question [32,33]. One highly cited review defines cumulative culture as ‘the ability of humans to ratchet up the complexity of cultural traits over time’ [31, p. 285]. This focus on complexity (see box 1 for definitions) is also found in Tennie *et al*.'s [26] landmark paper on the topic, where complexity is achieved by the normative dimension of cultural learning, which supports the extra faithful transmission of behaviour in humans. As already noted by Schofield *et al*. [46], this focus on complexity not achieved within one's lifetime has the consequence to exclude any non-human from the capacity for cumulative culture.

Box 1. **Complexity in the animal culture literature.**

Quantification of ‘complexity’ appear to be largely lacking in the animal culture literature. Generally, the term is invoked to describe intuitive and theoretical judgements regarding how ‘sophisticated’ a behaviour or its product is, or, perhaps less subjectively, the number of steps, elements and their combinations that are involved. For example, in the case of tool manufacture and use, researchers appear to use the term with reference to the number of procedural steps involved in the tool's production (e.g. [34]), the number of different objects combined and the syntactic structure of their combinations (e.g. [20]), the overall shape/structure of the final product (e.g. [5]), or the number of different functions the same tool is put to (e.g. ‘multifunction’ tools, [35]). Similar approaches are taken in the archaeological literature, where explicit attempts at measuring technological complexity involve counting the number of procedural units involved in a tool's production, i.e. the ‘minimum information that is needed to manufacture a product’ [36, p. S398], or quantifying a finished tool's distinct structures as ‘techno-units’ [37]. In biology more broadly, complexity is often equated with the number of different types of component units (e.g. cell types within a body, behaviour types in an animal's repertoire, relationship/interaction types within a species' social networks, number of species within an ecosystem [32,38–40]). Similarly, overall cultural complexity can be assessed on the basis of the range of different cultural behaviours detected within a species (e.g. [2]). While all of these approaches suffer potentially from complications associated with, for example, lumping versus splitting, they provide useful rules of thumb for at least qualitative comparisons.

In a systems or informational approach, complexity correlates with randomness, or unpredictability, in the sense that the more disordered the system is the more information it takes to define that system (e.g. Shannon entropy [41]). This definition is potentially compatible with definitions of complexity such as behavioural or toolkit diversity, as it is possible to consider the number of behaviours or tools, plus their usage frequency over some time interval as a macrostate, upon which an entropy measure could be taken. However, this too contains the problem of lumping versus splitting, as a vector of types must be defined by the researcher in order to calculate entropy. It could lead to the counterintuitive, and perhaps uninformative conclusion that, if two populations use the same two tools, but population 1 uses one tool more frequently, and population 2 uses both tools with a more balanced frequency, then population 2 is more ‘complex’ in an informational sense. Such an informational definition may also appear counterintuitive from a behavioural analysis perspective, where a behaviour with clear, multilevel rules is typically considered ‘complex’, whereas a more random sequence of actions would be considered less complex.

Over the course of evolution, we see increases in complexity in both morphology and behaviour—but more as an emergent property rather than a target of selection in its own right [42]. An example of evolution towards such acquired complexity concerns the shift from unicellular to multicellular life, this step itself leading to the emergence and refinement of a communication system between individual units of the same organism. Some potential exceptions include sexual selection, where trait complexity itself has, in some cases, been argued to be the target of selection. For example, in frogs, spiders and songbirds, mating signal complexity has been shown to be driven by receivers' sensory/cognitive biases for complex signals as these enhance the signals’ detectability and memorability [43–45].

In the light of increasing evidence of cultural change in animal species, the most recent attempt by Mesoudi & Thornton (M&T) [47] to consolidate the discussion in a more inclusive manner has divided the criteria for CCE into two categories, core and extended criteria. The core criteria include: (i) a change in a trait, (ii) the social transmission of that modified trait through a population, (iii) a realized increase in performance because of that trait, and (iv) that these three steps repeat. The authors hold that an increase in performance has consequences on inclusive genetic fitness, but also on ‘cultural fitness' when change in performance leads to increased wealth and/or material status. An example of the core criteria might be the sequential improvements in flight performance found over generations of homing pigeons derived from straighter flight paths [48], likely to increase fitness by saving energy and time, and reducing the risk of predation. The extended criteria include aspects of the more complex and open-ended cultural traits found in humans, such as chaining of multiple traits, diversification, recombination between cultural lineages, cultural exaptation and cultural niche construction [49].

With this paper, we extend this recent framing of CCE by focusing on performance in terms of efficiency, as efficiency is the most common focus in animal cultural evolution studies, especially in species where tool use is absent. From an exploration of the literature, we show how efficiency can be framed from the perspective of the organism (in terms of genetic fitness) as well as from the perspective of the behaviour itself (offering a definition of cultural fitness as learnability). In doing the latter, we acknowledge that we differ from M&T's cultural fitness, and instead align with the view adopted in studies of language evolution and cognitive science. We also extend the concept of informational bottleneck of social transmission from the evolutionary linguistics and cognitive science literature to the debate on CCE across species, and identify constraints that influence this bottleneck in wild populations. We argue that increases in efficiency allow traits to be more learnable and thus, more likely to persist when passing through the bottleneck of social transmission. We finally discuss how efficiency offers a pathway for the evolution of complex cultural traits.

## 2. Evidence for cultural evolution in animals

Beyond evidence of social learning and culture in various taxa [11–19], the last decade has produced increasing evidence for changes in the form, distribution or function of cultural traits in response to Darwinian-like processes such as drift or selection [50]. Both experimental manipulations and observational studies have produced a growing number of potential examples of cultural evolution in animals that meet the core criteria of CCE laid out by M&T [47].

In one potential example of the cultural evolution of foraging behaviour towards increasing complexity, New Caledonian crows (*Corvus moneduloides*) use three pandanus tool designs: wide tools, narrow tools and stepped tools [5]. These designs have been argued to be cumulative evolution from a common origin, with the more efficient tools evolving from less efficient ones. However, the higher efficiency of stepped tools was inferred from the apparent complexity of the tool, an assumed co-occurrence of complexity and efficiency. This assumption was tested explicitly in central African chimpanzees: their frayed stick tools used to fish for termites have experimentally been shown to be more efficient (i.e. retrieve more termites per dip) than non-frayed tools [34]. A possible reconstruction of their evolution posits that individuals modified original non-frayed tools to produce the more efficient frayed variant. Finally, even the earliest report of a putative cultural behaviour in animals—sweet potato washing by Japanese macaques [51]—has undergone change over the six decades since its first appearance. The gradual addition of novel steps to the behaviour is argued to represent ‘increases in complexity and efficiency of the washing behaviours' [46, p. 119].

One recent study was able to follow cultural evolution for efficient foraging behaviour in real time. In 2014, Hobaiter *et al*. [52] described the innovation and spread of a novel behaviour, moss-sponging, in the chimpanzees of Budongo Forest, Uganda. This new tool-using technique apparently originated from widely present leaf-sponging behaviour [53], and further work showed that the new technique continued to spread through social learning, most likely because moss tools are faster to manufacture while offering increased absorption capacity [54,55]. Finally, a recent experimental study found a positive relationship between population turnover and the cultural evolution of efficiency in great tits [56]; knowledgeable residents more often innovated an efficient alternative foraging behaviour, which was then adopted by less experienced immigrants, so that population turnover greatly increased the probability that this efficient behaviour invaded populations. This result echoed an older study in blueheaded wrasse, in which naive individuals better re-sampled options for mating sites compared with experienced, resident fish [57].

In an example of cultural evolution in the navigational domain, pairs of homing pigeons increased the efficiency of their socially transmitted travel routes across experimentally created ‘generations’, replicating results from transmission chain studies in humans [48,58]. Similarly, using observations of the gradual re-establishment of migratory routes in reintroduced species, migration paths of ungulates and whooping cranes were not only culturally transmitted, but also changed over time [59,60]. In both taxa, migration routes became more efficient over repeated flights (in cranes), or over generations (in ungulates), suggesting that the behaviour was being shaped by experience and innovation. Indeed, the weight of recent evidence suggests that migration routes and choice of stop-over sites may be shaped by generations of learners across many social species.

Song in many bird and mammal species is often socially learned from parents or neighbours. There is an extensive literature studying cultural evolution in song in passerine birds and cetaceans, far beyond what can be reviewed here. While much of this has focused on changes in the spatial distributions of song types (e.g. in the emergence or loss of local dialects), one recent example evidences increases in complexity in humpback whale song (*Megaptera novaeangliae*) [61]. This increased complexity is perhaps driven by female choice or male–male competition, but appears to be bounded, punctuated by regular decreases in complexity associated with the introduction of entirely new song types. Cultural evolution in passerine song can also be driven by environmental pressures on signal–receiver dynamics, arguably a form of efficiency. In the best-studied example, low-frequency traffic noise in cities has resulted in cultural evolution for higher frequency song across a multitude of bird species [62,63], and such cultural evolution allows socially learning species to exhibit a tighter noise-frequency adjustment [64].

The evidence summarized above highlights that much of cultural evolution in non-humans is characterized by the adoption of a more efficient behaviour compared to its ancestral version. This matches the requirement of increased performance in the core criteria of CCE: straighter travel paths [48], higher foraging rates [34,55] or better communication [65] as proxies for fitness. However, solely focusing on how behaviours improve genetic fitness does not capture the full answer of why distributions of cultural traits should change. In the following section, we review how efficiency has been discussed in the literature, and suggest that changes to efficiency can improve not only genetic fitness, but also the cultural behaviour's fitness, or learnability, which has often been neglected in the study of animal culture.

## 3. Defining and quantifying efficiency

One striking feature of the literature on cultural evolution is the myriad ways in which efficiency has been defined and quantified. To incorporate efficiency into the wider discussion on CCE, we reviewed the literature to identify animal cultural evolution studies in which efficiency was quantified, summarized in table 1. Overall we identified two broad categories for definitions of efficiency, either from (i) the organism's perspective, which generally involve metrics that might be apparent to the animal, such as latency to reward or energy investment, or (ii) the behaviour's perspective, which calculates efficiency by measuring the information contained by the behaviour itself. These two perspectives are not mutually exclusive, and overlap in certain cases. At the end of the section, we synthesize the two into a fundamental definition of efficiency that is summarized by a basic relationship between cost and benefit of a particular behaviour, that is efficiency ∝ benefit/cost.

**Table 1.** Review of definitions of efficiency in the literature on cultural evolution. The efficiency of socially transmitted behaviours has been defined in many ways over the past few decades. Examples are summarized here from studies which used non-human species, either exclusively or non-exclusively. Highlighted are the general categories of behaviour, the form of reward or measure, the definition of efficiency and the perspective of the definition.

perspective	study species	behaviour	efficiency definition	citation
organism	blueheaded wrasse (*Thalassoma bifasciatum*)	mating-site preference	increasing reward (predation protection)	Warner [57]
organism	great tit (*Parus major*)	foraging puzzle	increasing reward (food item)	Aplin *et al*. [66]
organism	New Caledonian crow (*Corvus moneduloides*)	extractive tool foraging	latency to reward (food item)	St Clair *et al*. [67]
organism	chimpanzee (*Pan troglodytes*)	extractive tool foraging	latency to reward (tool manufacture)	Lamon *et al*. [55]
			increasing reward (liquid absorption)	
organism	great tit (*Parus major*)	foraging puzzle	latency to reward (food item)	Chimento *et al*. [56]
organism	bighorn sheep (*Ovis canadensis*) moose (*Alces alces*)	migration routes	increasing reward (food availability via knowledge of phenology)	Jesmer *et al*. [59]
organism and behaviour	chimpanzee (*Pan troglodytes*)	extractive tool foraging	increasing reward behavioural structure (bimanual coordination)	Humle & Matsuzawa [68]
organism and behaviour	chimpanzee (*Pan troglodytes*)	extractive tool foraging	latency to reward behavioural structure (no. of hits)	Luncz *et al*. [69]
organism and behaviour	black rat (*Rattus rattus*)	foraging	energy invested (O_2_ consumption)	Terkel [70]
			latency to reward (food item)	
			systematic structure (stripping versus shaving)	
organism and behaviour	New Caledonian crow (*Corvus moneduloides*)	extractive tool foraging	increasing reward (tool useability) behavioural structure (tool structure)	Hunt & Gray [5]
organism and behaviour	Japanese macaque (*Macaca fuscata*)	foraging	less time and energy invested, reduced risk; behavioural structure (addition of novel acts)	Schofield *et al*. [46]
behaviour	zebra finch (*Taeniopygia guttata*)	song	behavioural structure	Feher *et al*. [71]
behaviour	homing pigeon (*Columba livia*)	flight route	behavioural structure (flight path straightness)	Sasaki & Biro [48]
behaviour	baboon (*Papio papio*), human children (*Homo sapiens*)	foraging puzzle	systematic structure (tetromino)	Claidiere *et al*. [7]
				Saldana *et al*. [72]

Crucial to M&T's definition of CCE is the increase in ‘performance’ derived from a cultural variant, which is a proxy for either genetic fitness (e.g. direct or indirect reproductive success) or cultural fitness (e.g. wealth, or social status). However, this view of cultural fitness does not integrate an alternative interpretation of cultural fitness as the ‘reproductive success' (transmissibility) of a trait, either through increases in learnability or its alignment with cognitive biases [73–75]. We favour the latter understanding of cultural fitness, as this distinction between genetic and cultural fitness sets up a definition of efficiency that takes into account both types.

### (a) Quantifying efficiency from the perspective of the organism

From the perspective of the animal, efficient behaviours that yield a higher benefit, or lower cost, should increase fitness. In a foraging behaviour, increasing benefits might arise from increasing the number of food items obtained by the behaviour, or the quality of food items [5,59,66,68]. In the case of mating-site preferences or bird song, benefits will probably be increased reproductive success [57]. Decreasing costs might come from reduced latency from the start of the behaviour to the reward [55,56,69], or decreased energy expenditure, measured, for example, in terms of oxygen consumption [70].

The ability to evaluate such pay-offs and costs should occur in a broad range of species without need for complex cognitive processes. Many animals can indeed recognize a more valuable pay-off, particularly during food-based tasks [76]. Individual reinforcement learning involving motivational cues and reward criteria [77] can work to keep a trait in the individual's behavioural repertoire, or lead to its abandonment [78]. This process allows for (i) selection between alternative variants, and (ii) potential modification of variants to increase reward rates. Behavioural conservatism or functional fixedness [79] are serious barriers to this pressure for efficiency in animals, as learning a new variant may increase costs in the short term, potentially preventing the switch to a behaviour which would yield greater reward rates in the long term. Animals that can cognitively assess and compare techniques (e.g. [55]), through extended representative and meta-representative abilities (e.g. allowing representing beliefs, such as in theory of mind, or mental time travel), might be more likely to pay the cost of learning a novel variant [80,81]. The demographic process of population turnover can also provide a path around behavioural conservatism without the need for such cognitive flexibility [56,57]. Regardless of the mechanism, from an ultimate perspective, individuals that adopt more efficient cultural traits may realize increased reproductive success. If there is vertical transmission of cultural behaviours, their offspring might acquire this more efficient behaviour, simultaneously inheriting its fitness benefits and promoting selection for that behaviour.

### (b) Quantifying efficiency from the perspective of the behaviour

Behaviours themselves may exhibit a quantifiable change in efficiency that increases their effects on genetic or cultural fitness. Here, we identify a type of informational efficiency which has often been termed ‘systematic structure’, which describes the amount of information needed to encode a behaviour, or set of behaviours. Because behaviour can be described at different levels of organization, (e.g. syllables, phrases and motifs in birdsong), this type of efficiency can be found at any given level of analysis.

Within individuals, behavioural efficiency can develop through individual trial-and-error learning, where repeated production over time reduces behavioural variation and noise. For example, an experienced learner might wield a novel extractive foraging tool better than a naive learner, displaying a more consistent, structured use of the tool. This could be quantified by measuring the movement of the tool through space, or the tracking of body posture through space as the tool is used. However, it remains an open question as to whether such increases in efficiency through individual learning matter for social transmission. Skilled demonstrators might be better demonstrators, resulting in fewer observations needed to learn to the behaviour. Alternatively, social learners that acquire behaviours from more skilled demonstrators might be initially more efficient, especially if imitation is the mechanism of learning. Such selectivity/bias in choice of models has been hypothesized as a possible social learning strategy [82,83], although empirical evidence in animals is scarce. For example, Ottoni *et al*. [84] have shown that capuchin monkeys attend to more skilled tool-users, although it is still an open question whether this improves observers' learning.

Between individuals, behavioural efficiency might also increase through iterated learning, either because behaviours themselves change to become more learnable or perceptual/production biases are amplified over generations of learners [74]. There is a large body of work on systematic structure with respect to language (discussed in box 2). More broadly, rules for or constraints on the production of the sub-behaviours within behavioural sequences constitute a syntactic grammar of behaviour (e.g. [100,101]). Some types of grammars are more compressible than others, are, therefore, more learnable, and thus more likely to be transmitted to the next generation of learners [73]. Critical to this pressure for learnability is an informational bottleneck between demonstrators and observers. There has been some application of this concept in the study of animal behaviour [102]. In animal communication, Menzerath's Law states ‘the greater the whole, the smaller the parts'. A recent comparative study has demonstrated this principle in bird song [96], although the authors suggest that this is owing to motor production biases rather than a result of informational bottlenecks. Menzerath's Law is also increasingly described in primate gestural and vocal communication [97–99]. The development of structure through iterated learning has been experimentally shown in zebra finch song, and argued to be a result of the amplification of perceptual or production biases [71,103]. Finally, systematic structure, alongside increasing task performance, has been shown to have evolved in a cultural transmission experiment run with baboons (*Papio papio*) [104].

Box 2. **Relevance of language and iterated learning for animal behaviour.**

One active area of research that informs the wider discussion of the evolution of efficiency and structure is the iterated learning paradigm, especially with respect to the evolution of language. This body of literature, well supported by mathematical and computational models, and experimental work [74], was originally concerned with questions relating to the origin of structure in language, but has since branched out to cover more behavioural domains, such as music [85–87]. Broadly, this body of work emphasizes the importance of the informational bottleneck for the cultural evolution of systematic structure, and the amplifying effect that iterated learning has on prior biases. This bottleneck has also been highlighted in the cognitive science literature; this has been explored as the ‘now-or-never’ bottleneck [88].

Language has been argued to be an adaptive system that balances efficiency and complexity [73]. It is transmitted through iterated learning: the repeated observation, hypothesis inference and subsequent production of language, typically during an extended period of development. It has a systematic, compositional structure at multiple levels of organization, in which the meaning of a complex signal is a function of its components [89]. The complexity of language is ultimately constrained, as realized human languages are quite optimized to balance trade-offs at various levels of organization [90,91]. One of language's primary functions is to express the immeasurably complex world around us [92]. This requirement of expressivity provides a pressure for potential complexity. However, the phonetic, morphemic and syntactic information that comprises language must pass through the informational bottleneck of social learning [73]. This bottleneck has been proposed to provide a cultural evolutionary pressure for more efficient, compositionally structured grammars of language, as structured information is compressible, easier to learn and pass through the bottleneck, and thus more likely to persist in the next generation of users [93,94].

Of course, no systematic behaviour at the scale of language has yet been found in animals [95]. However, every socially learned behaviour must pass through an informational bottleneck. Thus, animal culture, communicative or otherwise, should be under a similar pressure for learnability. While experimental work is scarce, this is supported by evidence of Menzeranth's Law in animal communication systems [96–99]. Non-communicative animal behaviour lacks a pressure for expressivity to stave off degeneration, but there are plenty of other pressures for maintaining complexity, as behaviours must still generate pay-offs for an animal to remain in their repertoire.

Overall, systematic structure is understudied in animals. Whether systematic structure of animal culture changes over time, either owing to pressures for learnability or alignment with pre-existing biases, will come from future studies which quantify changes in informational complexity over generations of learners. This begs the question of how to quantify learnability, or informational structure, within a behaviour. One broadly useful method is the analysis of ethograms using hidden Markov models, such as in a recent study comparing the information content of Acheulean and Oldowan tool manufacture [105]. This allows for comparison of information content, as well as compressibility (a proxy for learnability), between behaviours.

### (c) Integrating the two perspectives

Our organismal and behavioural perspectives might seem distinct, but can be connected to each other by the basic relationship between cost and benefit of a particular behaviour, that is efficiency ∝ benefit/cost,  where benefits are those accrued from producing a behaviour, and costs include the cost of both learning and producing the behaviour. This relationship can further be expanded to:

$$\mathrm{efficiency}\;\propto \;\frac{\sum \pi }{C_{A}+\;\sum C_{P}}.$$

From this relationship, the efficiency of a particular behaviour could rise by increasing benefits, here defined as the cumulative sum of all received pay-offs over the lifetime of the individual (∑π). These benefits are considered against their attendant costs: the cost of acquisition (*C_A_*), and the sum of all costs of producing the behaviour $\left(\sum C_{P}\right)$.

Efficiency from the perspective of the organism can describe these sum benefits and costs of production. It is readily apparent how the number of food items received from a behaviour, or the physical exertion required to execute a behaviour would affect these terms, as these are immediate costs and benefits to the organism upon behavioural production. For example, the migratory path of an ungulate should change over generations of learners to better follow the green wave of plant phenology to maximize reward [59], yet should also change to avoid difficult environmental barriers that would increase exertion. Efficiency from the perspective of the behaviour can also describe the benefits and costs of production. Indeed, there may be many examples when a more structured behaviour aligns with the definition of efficiency from the organism's perspective, either decreasing latency to reward through refinement of a behaviour via individual learning [56]. For example, structured flight paths that incorporate a specific memorized landmark sequence may lead to shorter homing times [58], or a specific sequence of actions during nut-cracking might lead to fewer tool strikes being needed to open a nut.

Within the literature on animal culture, the most neglected term in this equation is the cost of acquisition, and how it might be affected by the efficiency, or systematic structure, of a behaviour. Increased systematic structure should reduce the cost of acquiring the behaviour, as it has a concomitant reduction in its encoding complexity, and improves learnability. This would have the important consequence of increasing a behaviour's cultural fitness, e.g. by reducing the likelihood of extinction through turnover, or other information loss. Further, any energetic costs saved while learning a behaviour might be reinvested elsewhere. The cost of acquisition, or pressure for learnability, is determined by the bottleneck of social transmission, which we will discuss in the context of wild populations in the following section.

The analysis and integration of these definitions supports the growing consensus that efficiency underlies the core criteria for cumulative culture [47], and expands the original concept of performance beyond improvements in proxies for genetic fitness, or social status and material wealth. Structured behaviours might be more efficient for the animal themselves (increasing genetic fitness), but also might be more learnable by the next generation of learners (increasing cultural fitness, and genetic fitness). Meanwhile, the requirement of reward in the numerator of our equation prevents the evolution of maladaptive or degenerate (highly learnable, yet unrewarding) behaviours in populations of wild animals.

## 4. Efficiency and the information bottleneck in natural populations

**Figure 1.**
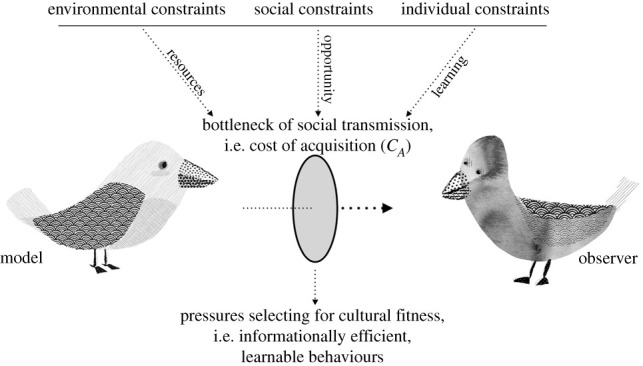
The bottleneck of social transmission in wild populations. The bottleneck of social transmission is usually thought of in the context of dyadic learning between humans, and is framed by individual constraints such as memory and attention. However, the size of the bottleneck should take into account the summed opportunity for potential transmission, as this affects the overall amount of memory available at the population level. Opportunity can be influenced by environmental constraints such as the distribution of resources, as well as social constraints, such as dominant/subordinate relationships. Behaviours that must pass through a stricter bottleneck should be under pressure to become more learnable, or face behavioural extinction.

In the previous section, we reviewed how efficiency results from the relationship between benefits and costs of production. In this section, we highlight the cost of acquisition for animals, and how it relates to the bottleneck of social transmission (figure 1). As a metaphor, a bottleneck implies that a large volume must pass through a constraint. In biology, it has often been used as a metaphor when populations experience a reduction in size, such as during the colonization of an island. In the literature on iterated learning, the term has been used to describe reconstruction of a language by naive learners through the observation of linguistic input. This informational bottleneck is typically framed within an abstracted concept of dyadic transmission of language between humans, and is constrained by memory limitations [74,88,106].

We pick up on this definition, and suggest that all socially transmitted behaviours must pass through an informational bottleneck and be reconstructed on the other side. In addition to individual-level constraints such as memory, the size of the bottleneck should take into account the summed opportunity for social learning over a given period of time, as increased opportunities for transmission increases the available ‘memory’ in a population, and similarly relaxes pressures for learnability. In this section, we give an overview of constraints which might affect the opportunities and direction of social learning in wild populations of animals, including environmental, social and individual constraints. All may ultimately affect underlying probabilities of transmission of a particular trait, and thus its survival. When the bottleneck is more severe, more learnable traits have a better chance of persisting in wild populations. This universal pressure for learnability, or improved cultural fitness, should be considered alongside the traditional proxies for genetic fitness in CCE.

### (a) Environmental constraints

Environmental constraints can greatly increase the cost of acquisition of a behaviour by altering the likelihood of innovation or transmission. Object-based traits such as tool-using behaviours may be especially constrained, as they involve at least three steps (tool making or acquisition, finding the resource and using the tool) [107]. Reversely, the changing environment might provide opportunities for cultural evolution [108]. For example, in a case of cultural exaptation, Luncz *et al*. [109] found that long-tailed macaques transferred their stone-tool use for harvesting shellfish to additionally cracking nuts at abandoned oil-palm plantations, thus broadening the range of accessible resources. The recent heated debate on the extent to which ecological variation influences cultural variation in animals [2,110] has led to multiple studies of ecological correlates of animal tool use over the last two decades [111–117]. These studies have demonstrated that the two major factors which mediate the use of tools are opportunity (encountering tool material and substrate for tool use with sufficient frequency in one's environment), and necessity (e.g. periods of food shortage that trigger tool use as an adaptive response). While studies have typically found more support for one or the other [111–114], it is likely that both factors interact to cause tool use [115,116], possibly following an adaptive rule of profitability, with tool use being favoured when it is the most rewarding strategy [117]. An example of this is found in faster manufacture and/or use time, as in the case of moss-sponges in wild chimpanzees [55]. The probability of innovation of a more efficient behaviour itself depends on the environment, which might not provide a more efficient substrate (e.g. moss, or frayable sticks) from which to innovate the new tool, increasing costs of innovation. Overall, efficiency provides an incentive to use particular resources, nesting or foraging sites, but it will be dependent on the properties of this environment, as well as their potential for change that results in increased pay-offs.

### (b) Social constraints

The social environment constrains learning opportunities, as well as the direction of transmission, thus affecting the overall size of the bottleneck. There is a large literature, particularly in the field of human cultural evolution, which highlights the influence of various network properties such as population size, structure and dynamic turnover on the emergence and evolution of cultural traits (see [56] for a review). For example, larger populations have both an elevated probability of innovation and faithful cultural transmission [118–121], while population reduction, either through translocation or colonization, can result in a loss of diversity and simplification of traits, as observed in bird song [122–124], resulting from founder effects and population bottlenecks [123,125,126]. However, these findings contrast with increasing variation and loss of regular structure found in song syntax found in island populations of chaffinches [127]. This example was argued to be a result of a cultural trap, where relaxed learning biases evolved as a result of receivers benefitting from recognizing all potential conspecifics. Further, syntactic structure might itself be under sexual selection in continental populations, and might not be as important once this selective pressure is relaxed in founding populations. In such a case, the cost of acquisition of novel, perhaps more difficult to learn innovations might be reduced, as benefits remain stable between syntactically regular and irregular song.

Population turnover causes faster memory loss across a population, and thus a smaller overall bottleneck. When knowledgeable individuals are replaced by immigrants or new generations, this has two effects: (i) the pool of demonstrators is reduced, and (ii) established behaviours are weighted less strongly. This has been argued to enable a population of individuals to better resample their behavioural space, increasing the likelihood that novel, more efficient (from the perspective of the organism) traits invade the cultural repertoire [56,57]. Turnover should also increase pressure for learnability, as the faster turnover occurs, the less opportunity there is for transmission from knowledgeable to naive individuals. If turnover is too fast, and the cost of acquisition is too high, cultural traits could become extinct. Related to this, age structure of populations is likely to be critical. For instance, if older, more experienced, individuals are targeted for hunting, this will decrease opportunities for observation, and remove the benefits of a lifetime of individual reinforcement learning on socially learned traits. For example, in both crane and cod migration routes, removing older individuals led to less straight lines [60].

In addition to these population-level social factors, dyadic social interactions can influence the bottleneck. For example, any form of demonstrator bias constrains the opportunity of social transmission [128]. In primates, for example, dominant individuals are often preferred demonstrators, limiting the set of transmittable behaviours to those individuals' repertoire. Further, dominant and subordinate interactions might increase the cost of acquisition, especially if multiple observations are required to acquire a behaviour. A subordinate individual might struggle to remain in the vicinity of a dominant individual who is monopolizing a food source, making exposure to the new behaviour less likely. If a more structured behaviour is easier to learn, it may necessitate fewer observations in such a social environment. However, this can be compensated for by learning through kin, rather than through other conspecifics [54].

### (c) Individual constraints

In addition to the above, individual-level factors can also affect the informational bottleneck of social transmission. First a better, larger memory widens the bottleneck, allowing for more detailed behavioural information to be remembered after observation. Oscine birdsong, a cultural behaviour which has a high observed fidelity, has been shown to rely on life-history specific changes to the faculty of memory during the critical period. It has long been recognized that juvenile birds learn many more songs than they will use as adults, and their knowledge undergoes attrition, resulting in a subset of syllables used in adult song [6,129]. The enhanced ability to learn a large repertoire in a short period, combined with overproduction and attrition, leads to incredibly stable traditions [130], and might limit pressure for song learnability. Interestingly, a better memory might promote behavioural conservatism, impeding the cultural evolution of more efficient behaviours, as individuals are more likely to remember pay-offs for previous behaviours and less likely to sample new behaviours. Novel behaviour must compete with known behaviour, and often it is safer to perform well-honed behaviour, rather than a novel behaviour in a risky environment, as already known traits offer a local optimum, in terms of ease of use and pay-off.

Another individual factor with a similar effect as memory on the bottleneck is attention. In the wild, animals must divide their attention across many cues at all times. The amount of attention that can be invested in social cues is, therefore, a constraining factor, as relatively undivided attention is required for the most accurate observation of a target behaviour. In addition, natural social learning occurs in a socio-emotional/physiological context that also has potential implications on learning in naive individuals [131]: a stressed individual might not be as attentive to the details of a given innovation compared to a hungry or satiated one, threatening the transmission of information altogether.

Lastly, there might be individual variability in causal understanding of whether a novel innovation is more beneficial, making adopting that innovation more complicated for some individuals compared to others. While it has been shown that human cumulative culture does not need to be causally understood ([132], but see [133]), the ability to represent one's and others’ knowledge remains an important factor in the very existence of cultures [80]. While not mandatory, causality is likely to be part of the discussion, especially considering that the most likely candidates for CCE in animals are found in species considered as creative tool-users [134] that probably have some kind of representational access to their tools, and possibly to their cultures [80,81].

## 5. Conclusion

Definitions of CCE that favour complexity are still present throughout the anthropology and psychology literatures [135]. As a result of these framings of CCE, human cultures are seen as highly dynamic, while to the contrary, the study of animal cultures has often been limited to analysing behavioural traits as static responses to the environment, with little cultural change observed or postulated. When cultural change has been shown, the use of the word cumulative to characterize these traits remains controversial for some authors (reviewed in [47,108]).

To bridge this divide, we propose that complexity is not possible without learnability, which is equivalent in our view to efficiency from the behaviour's side. The adoption of a complex behaviour represents a movement to a new local optima, with a greatly increased cost of acquisition alongside a concomitant increase in benefit. Multicomponent behaviours must be composed of easy-to-learn components if they have any hope of passing through the bottleneck of social transmission. For example, the grammars of Acheulean tool manufacture are more informationally complex, yet more compressible, and learnable compared to the Oldowan technique [105]. This is because their transmission becomes exceedingly difficult when the availability of information to learners is ephemeral in the environment, as learners have limited memory and attention, and can be influenced by a range of circumstances that will limit the probability of successful transmission.

Overall, the main goal of this paper is to identify efficiency as a point of consensus in CCE, and to develop the concept of efficiency in the light of the literature from the broad fields that fall under the study of cultural evolution. Cultural evolutionary pressures for efficiency, or increased benefit relative to cost, should be found across all species owing to clear realized benefits to either genetic or cultural fitness. Efficiency from the perspective of the organism might be recognized and actively selected for through reinforcement learning that favours more rewarding behaviours. From the perspective of the behaviour, behaviours might evolve to be more efficient because more efficient, informationally structured traits should be more learnable, and thus more likely to spread through populations. Overall, our framework of efficiency can be used and tested across species, and offers a new perspective on the evolution of cumulative culture that connects cultural change and the burgeoning evidence of CCE in non-humans to the more complex phenomenon evidenced in humans.

## Data accessibility

This article has no additional data.
